# RITA-T (rapid interactive screening test for autism in toddlers): a scoping review of psychometric properties, cross-cultural validation, and clinical applications

**DOI:** 10.3389/fpsyt.2026.1922791

**Published:** 2026-08-27

**Authors:** Huijuan Liu, Guiju Zhang, Ziyun Li, Ting Zhang, Li Zhang, Yan Li

**Affiliations:** 1 Clinical Research Center for Child Health and Disorders, Shandong Provincial Maternal and Child Health Care Hospital Affiliated to Qingdao University, Jinan, China; 2 The First Clinical Medical College of Shandong University of Traditional Chinese Medicine, Shandong University of Traditional Chinese Medicine, Jinan, China; 3 Affiliated Hospital of Shandong University of Traditional Chinese Medicine, Jinan, China

**Keywords:** autism spectrum disorder, cross-cultural validation, early screening, interactive assessment, RITA-T, toddlers

## Abstract

**Background:**

The Rapid Interactive Screening Test for Autism in Toddlers (RITA-T) is a clinician-administered, interactive screening instrument for toddlers aged 18–36 months that directly assesses core social-communicative abilities through structured play. Since its introduction in 2015, validation studies have emerged across diverse populations and cultural contexts.

**Objective:**

This scoping review systematically maps the evidence on RITA-T’s development, psychometric properties, cross-cultural validation, clinical applications, and comparison with other level 2 screening instruments.

**Methods:**

A systematic search of PubMed, Web of Science, Scopus, PsycINFO, and CNKI was conducted through June 2026. Studies reporting original data on RITA-T administration in toddlers aged 18–36 months were included. Methodological quality was assessed using the QUADAS-2 tool.

**Results:**

Of 48 records identified, 10 original studies met the inclusion criteria: 7 diagnostic validation studies encompassing 659 toddlers across five countries (United States, Canada, Lebanon, Turkey, and China) and 3 implementation or training studies. RITA-T yielded strong psychometric properties across settings, with reported cutoff scores of 14–17, sensitivity of 0.74–1.00, and specificity of 0.50–1.00. Cross-cultural adaptations demonstrated comparable diagnostic accuracy across English, Arabic, Turkish, and Chinese versions.

**Conclusions:**

RITA-T represents an effective level 2 ASD screening tool with robust cross-cultural applicability. Future research should address long-term predictive validity, telehealth adaptation, and cost-effectiveness.

## Introduction

Autism spectrum disorder (ASD) affects approximately 1 in 31 among 8-year-old children in the United States (1) and roughly 0.7%–1.0% of children in China (2, 3), with global prevalence estimated at approximately 1 in 100 children (4). Despite rising prevalence and expanded awareness, the median age of ASD diagnosis in the United States remains approximately 4–5 years, and only about 50% of children with ASD receive their first diagnosis by 48 months (5). Diagnostic delays are even more pronounced in underserved populations and low-resource settings (1).

Core features of ASD (persistent deficits in social communication and interaction, alongside restricted and repetitive patterns of behavior) are reliably identifiable from 18 months of age (6). The American Academy of Pediatrics (AAP) has recommended universal ASD screening at 18- and 24-month well-child visits since 2007 (6), a recommendation reaffirmed and expanded in the 2020 clinical guideline (7). Early diagnosis followed by intervention has been shown to significantly improve social, cognitive, language, and adaptive functioning outcomes (8–10). The Early Start Denver Model (ESDM), for example, has demonstrated sustained gains in IQ and adaptive behavior in randomized controlled trials (9, 10), although a recent European multi-site randomized trial found no significant benefit of ESDM over treatment as usual on the primary developmental outcome (11).

Despite the recognized importance of early detection, significant barriers persist. Most widely used screeners, such as the Modified Checklist for Autism in Toddlers, Revised (M-CHAT-R/F) (12), rely on caregiver report, which is subject to caregiver-report bias, variable health literacy, and cultural differences in interpreting developmental milestones. Large-scale implementation studies have shown that first-level screening tools typically yield moderate positive predictive values (PPV), generating high rates of false positives that strain diagnostic resources (13). Real-world evidence from electronic medical records further indicates that screening questionnaires in routine practice have lower PPV than efficacy trials suggest (14, 15). Cross-cultural adaptations of the M-CHAT have been conducted in numerous countries, including Spain (16, 17), Albania (18), Chile (19), and Malaysia (20), with variable psychometric performance across settings. Briefer instruments such as the Quantitative Checklist for Autism in Toddlers (Q-CHAT) have also been developed for large-scale population screening (21). All questionnaire-based tools, however, share the inherent limitation of relying on parental observation and report.

A complementary approach focuses on directly assessing early social-communicative markers of ASD through interactive, performance-based tasks. Deficits in joint attention (the ability to coordinate attention between a social partner and an object or event) are among the earliest and most reliable behavioral markers of ASD in toddlers (22). Impairments in joint attention initiation, response to name, social referencing, and agency awareness can be elicited and measured through structured observation rather than parent report (22–24).

Disparities in ASD diagnosis persist across racial, ethnic, and socioeconomic lines. Black and Hispanic children with ASD without intellectual disability are significantly less likely to be detected than White peers (25), and non-White and low-socioeconomic-status children receive diagnoses significantly later (26). Systemic barriers, including provider bias, cultural mistrust, and resource gaps, contribute to these disparities (27). An interactive, performance-based tool may mitigate some of these disparities by reducing reliance on parent report and cultural interpretation of developmental milestones. The substantial economic costs of ASD across the lifespan (28) underscore the importance of early detection: universal screening at 18 and 24 months has been shown to be cost-effective compared with surveillance monitoring alone, yielding earlier diagnosis and lower lifetime costs per quality-adjusted life year gained (29).

The Rapid Interactive Screening Test for Autism in Toddlers (RITA-T) was developed to address these gaps. Unlike questionnaire-based instruments, RITA-T employs direct observation and structured interaction to elicit and measure key social-communicative behaviors.

## Methods

This scoping review followed the Preferred Reporting Items for Systematic Reviews and Meta-Analyses extension for Scoping Reviews (PRISMA-ScR) guidelines (30). The PRISMA-ScR checklist is provided as a Supplementary File. The review was not prospectively registered, and no review protocol was published prior to commencement. The eligibility criteria and data extraction items were predefined based on the PICOS framework and the PRISMA-ScR checklist, and the complete search strategy, eligibility criteria, and data extraction framework are provided as **Supplementary Materials**.

### Search Strategy

A systematic literature search was conducted in PubMed, Web of Science, Scopus, PsycINFO, and the China National Knowledge Infrastructure (CNKI) from database inception through June 2026. The search strategy combined terms for the target instrument (“Rapid Interactive Screening Test for Autism in Toddlers” OR “RITA-T”) with terms for autism screening (“autism” OR “autism spectrum disorder” OR “ASD” OR “screening” OR “early detection”). Reference lists of included studies and related reviews were hand-searched for additional records. The full search strategy for PubMed is detailed in **Appendix A**.

### Inclusion and exclusion criteria

Studies were included if they (a) reported original empirical data on RITA-T administration in toddlers aged 18–36 months, (b) included a comparison with a reference standard (ADOS-2, ADOS-G, DSM-5 clinical diagnosis, or multidisciplinary team evaluation), (c) reported at least one psychometric property (sensitivity, specificity, predictive values, area under the curve, or reliability coefficients), and (d) were published in English or Chinese. Studies were excluded if they were review articles, conference abstracts, case reports, or did not report original RITA-T data.

### Study selection and data extraction

Two reviewers (HL and GZ) independently screened titles and abstracts, followed by full-text review of potentially eligible studies. Disagreements were resolved through discussion. A standardized data extraction form was developed and piloted on three randomly selected studies before full extraction. Two reviewers (HL and ZL) independently extracted data on study characteristics (author, year, country, sample size, age range), RITA-T administration details (cutoff score, training of administrators), psychometric properties (sensitivity, specificity, PPV, NPV, AUC, reliability), and reference standard used. Discrepancies were resolved by consensus or adjudication by a third reviewer (LZ). No quantitative synthesis (meta-analysis) was planned given the anticipated heterogeneity in study designs, populations, and outcomes; a narrative synthesis approach was adopted.

### Quality assessment

Methodological quality of included studies was appraised using the Quality Assessment of Diagnostic Accuracy Studies 2 (QUADAS-2) tool (31), which evaluates risk of bias across four domains (patient selection, index test, reference standard, flow and timing) and applicability in three domains. Two reviewers (HL and TZ) independently rated each study; disagreements were resolved through discussion.

## Results

### Study selection

**Figure 1:**
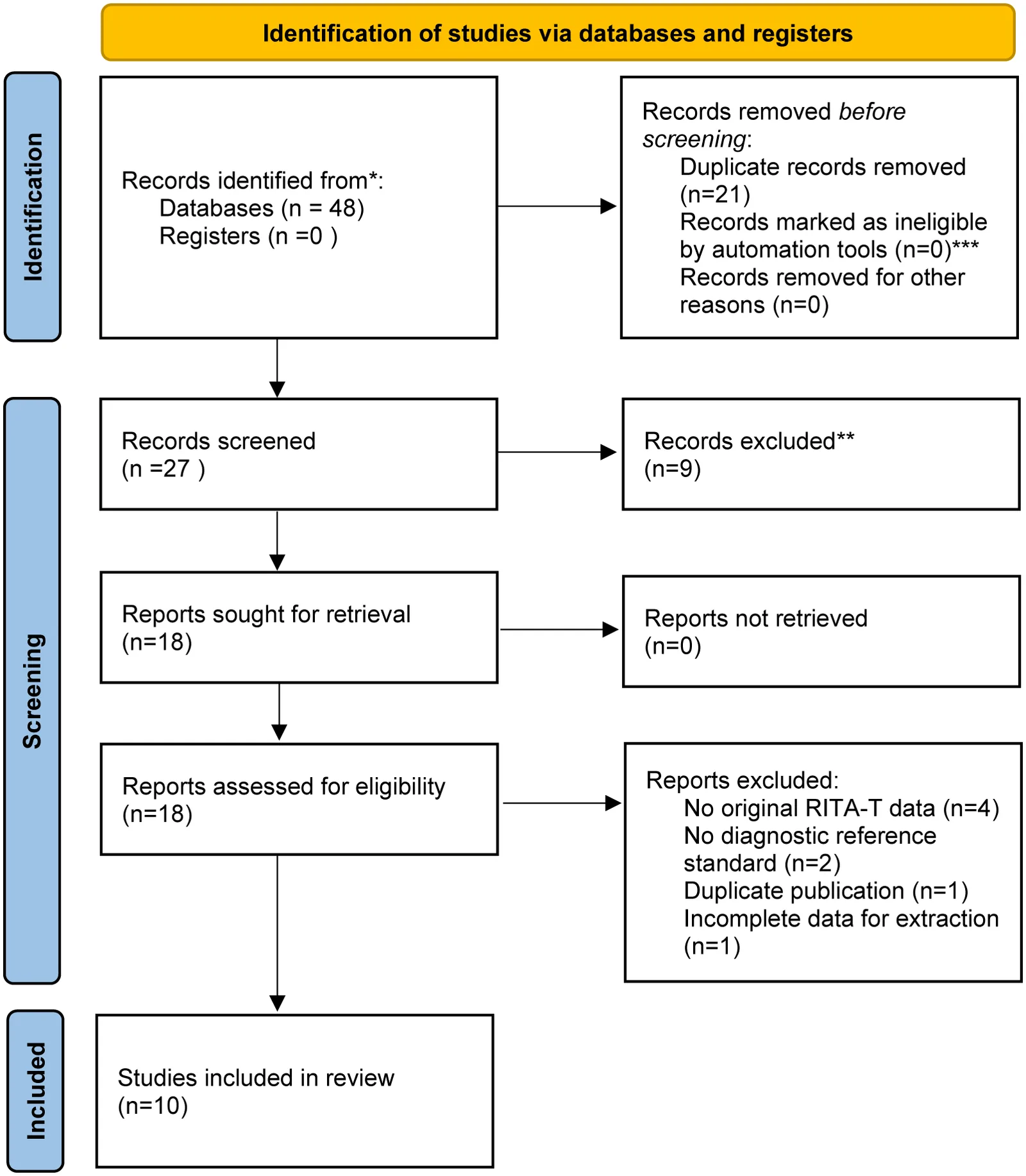
PRISMA 2020 flow diagram. *PubMed yielded 31 records; additional records were identified through Web of Science, Scopus, PsycINFO, CNKI, and hand-searching of reference lists. **Screening was performed independently by two reviewers (HL and YL); disagreements were resolved by consensus or consultation with a third reviewer (XZ). ***No automation tools (e.g., Al-based screening software) were used in this review.

The systematic search yielded 48 records across all databases. PubMed yielded 31 records; additional records were identified through searches of Web of Science, Scopus, PsycINFO, and CNKI, as well as hand-searching of reference lists of included studies and related reviews. After removing 21 duplicates, 27 titles and abstracts were screened; 9 were excluded as clearly irrelevant (reviews, editorials, or unrelated topics). The remaining 18 full-text articles were assessed for eligibility, of which 8 were excluded for the following reasons: no original RITA-T data (n = 4), no diagnostic reference standard (n = 2), duplicate publication (n = 1), or incomplete data for psychometric extraction (n = 1). A total of 10 original studies met all inclusion criteria and were included in this review. Manual searching of reference lists identified no additional eligible studies. The study selection process is detailed in the PRISMA flow diagram (**Figure 1**).

### Study characteristics

The 10 included studies comprised 7 diagnostic validation studies encompassing 659 toddlers across five countries (United States, Canada, Lebanon, Turkey, and China) and 3 implementation or training studies. Sample sizes in the diagnostic validation studies ranged from 35 to 239 participants. All studies used ADOS-2, ADOS-G, or DSM-5 clinical diagnosis as the reference standard. Five of the 10 included studies were authored or co-authored by the tool developer (Choueiri and colleagues) (32–36).

### Development and original validation

RITA-T was developed by Roula Choueiri and Steven Wagner at the University of Massachusetts Medical School and first described in 2015 (32). The test was designed as a level 2 screening tool for use following a positive level 1 screening result or when clinical concern for ASD exists. It targets children aged 18–36 months and requires approximately 10 minutes to administer.

The test comprises nine items that assess distinct social-communicative domains. Core joint-attention items include Phone Teasing, Line of Vision Obstruction, Magic Cup or Ball, Color-Changing Scarf, and Rapid Joint Attention Response. Social awareness is evaluated through Toy Phone Obstruction, Preference: Object versus Face, and Response to Caregiver Emotion. Agency, visual problem-solving, self-awareness, and communication are assessed by the remaining items. Each item is scored according to specific behavioral criteria using both timed and untimed components, yielding a total score ranging from 0 to 30; higher scores indicate greater ASD risk. Item selection was guided by established developmental markers of ASD as specified in DSM-5 (37), including deficits in joint attention, response to name, social referencing, and representational play.

In the initial validation study (32), Choueiri and Wagner administered RITA-T to 61 toddlers (aged 18–36 months) who were referred for autism evaluation or had no developmental concerns. The sample included 23 children diagnosed with ASD, 19 with other developmental disorders, and 19 typically developing children. A cutoff of 15 yielded sensitivity of 100% and specificity of 84%, with a PPV of 88% and NPV of 100%. RITA-T scores correlated positively with ADOS-G total scores, supporting convergent validity. Children with ASD scored significantly higher than both typically developing children and children with language delay or global developmental delay, indicating satisfactory discriminant validity.

### Psychometric properties

The reliability of RITA-T has been examined across multiple populations. The Chinese validation study reported a Cronbach’s alpha of 0.828, indicating good internal consistency (38). Test-retest reliability was high, with a Spearman correlation coefficient of 0.962 (p < 0.01). Inter-rater reliability, assessed via intraclass correlation coefficient (ICC), was 0.975 (95% CI: 0.970–0.979, p < 0.001), suggesting near-perfect agreement between independent raters (38). This finding is particularly relevant for clinical settings in which multiple healthcare providers may administer the test. The Chinese validation provided the most comprehensive reliability assessment across all three indicators, whereas the Arabic (36), Turkish (45), and Canadian (43) adaptations reported only limited reliability data, flagging a discrepancy that future cross-cultural studies should address.

Regarding validity, RITA-T items were developed based on established developmental markers of ASD in toddlers, and the nine items map onto core diagnostic domains specified in DSM-5 (37). Convergent validity is supported by significant positive correlations between RITA-T scores and ADOS-G or ADOS-2 total scores (32, 39). The ADOS-2 Toddler Module has been independently validated as a gold-standard diagnostic instrument in this age group (40–42). Kong et al. (39) evaluated RITA-T classification performance against ADOS-2 classification and DSM-5 diagnosis as separate reference standards in 35 individuals aged 18–84 months. When ADOS-2 classification was used as the reference standard, RITA-T achieved sensitivity of 0.81 and specificity of 0.89. When DSM-5 diagnosis was used as the reference standard, RITA-T achieved sensitivity of 0.74 and specificity of 0.50. Across studies, reported cutoff values range from 14 to 17, with sensitivity of 0.74–1.00 and specificity of 0.50–1.00, depending on the population and reference standard (32, 39, 43, 45). RITA-T has also demonstrated the ability to distinguish ASD from other developmental conditions. The original study (32) found that children with ASD scored significantly higher than children with global developmental delay or language disorders, and the Chinese validation further confirmed RITA-T’s capacity to differentiate ASD from global developmental delay (GDD) and developmental language disorder (DLD) (38).

In terms of structural validity, no included study reported exploratory factor analysis (EFA), confirmatory factor analysis (CFA), or model fit indices. EFA is a data-driven method that identifies latent factor structures without requiring *a priori* hypotheses, whereas CFA tests whether observed items conform to a hypothesized dimensional model. Model fit is typically evaluated using multiple indices: the root mean square error of approximation (RMSEA; values < 0.06 indicate good fit), the comparative fit index (CFI; values > 0.95 indicate good fit), and the Tucker–Lewis index (TLI; values > 0.95 indicate good fit), following established consensus criteria. Two factors account for the absence of these analyses in the current evidence base: first, the original RITA-T development literature (32) did not derive explicit theoretical dimensions, leaving subsequent validation studies without an *a priori* factor structure to test via CFA; second, validation studies focused primarily on diagnostic accuracy metrics without exploring the internal factor structure. Future validation work should first conduct EFA to identify the latent dimensionality of RITA-T’s nine items, then test the resulting structure via CFA, and assess cross-cultural measurement invariance.

### Quality assessment of included studies

Methodological quality of the 10 included studies was evaluated using the QUADAS-2 tool (31). Risk of bias and applicability concerns are summarized in **Table 1**:

**Table 1:** Quality assessment of included studies using the QUADAS-2 tool.

Study	Patient selection	Index test	Reference standard	Flow & timing	Overall applicability concern (author-derived summary)	Methodological flaw	Impact on results
Choueiri & Wagner (2015) (32)	High	Low	Low	Low	High concern	Selection bias (case-control)	Sensitivity/Specificity possibly overestimated
Yassin et al. (2020) (36)	High	Low	Low	Low	High concern	Selection bias (case-control)	Sensitivity/Specificity possibly overestimated
Lemay et al. (2020) (43)	Low	Low	Unclear	Low	Low concern	Consecutive referred cohort; low risk	Minimal bias concern
Choueiri et al. (2021) (33)	Low	Low	Low	Low	Low concern	Low risk	Minimal bias concern
Kong et al. (2021) (39)	Unclear	Low	High	Low	High concern	Review bias (examiners not blinded to screening findings); Applicability concern (participants aged up to 84 months)	Diagnostic accuracy estimates may be biased; applicability to toddlers aged 18–36 months limited
Choueiri et al. (2023) (34)	Unclear	Low	Unclear	Unclear	Unclear concern	Unclear risk (retrospective chart review)	Results should be interpreted with caution
Choueiri et al. (2024) (35)	High	Low	High	Unclear	Unclear concern	Potential review bias (clinician aware of RITA-T)	Prior knowledge of RITA-T results may have biased diagnostic accuracy estimates
Kadak et al. (2024) (45)	Unclear	Low	Low	Low	High concern	Unclear risk (retrospective design); applicability limited	Results should be interpreted with caution
Liu et al. (2026) (38)	Unclear	Low	Unclear	Low	Unclear concern	Reference-standard blinding not reported	Results should be interpreted with caution
Richter et al. (2026) (44)	Not applicable	Not applicable	Not applicable	Not applicable	Not applicable	Training/implementation study (not a diagnostic accuracy study); QUADAS-2 excluded	Not applicable to diagnostic accuracy evaluation

Risk of bias ratings reflect study design characteristics (e.g., case-control enrollment, absence of blinding) rather than failures in study conduct. The overall applicability concern is an author-derived summary, defined as the most conservative rating across the three QUADAS-2 domains that include an applicability assessment (patient selection, index test, and reference standard). The flow-and-timing domain does not include an applicability judgment in QUADAS-2. N/A indicates that the study was excluded from the QUADAS-2 assessment.

Note: Risk of bias ratings reflect study design (e.g., case-control design in early validations introduces selection bias) rather than study conduct. “High” risk in patient selection for early studies reflects the use of case-control or convenience sampling. Richter et al. (44) was excluded from the QUADAS-2 assessment because QUADAS-2 was designed for diagnostic accuracy studies, and this training/implementation study does not fit its domain structure. The overall applicability judgment for each study was derived as the most conservative rating across the three QUADAS-2 domains that include an applicability assessment (patient selection, index test, and reference standard), because the flow and timing domain does not include an applicability judgment. Because QUADAS-2 does not define a single overall applicability rating, this summary rating is an author-derived synthesis rather than a standard component of QUADAS-2 reporting: “Low concern” was assigned only when all applicable domains were rated Low, and “Unclear concern” or “High concern” reflected the most conservative rating across domains. A complete QUADAS-2 assessment, including the signaling questions for each domain and the domain-specific applicability judgments for each study, is provided in **Supplementary Table 1**.

Three recurring methodological flaws can be categorized by their impact on diagnostic accuracy estimates. First, selection bias: the two early case-control studies (32, 36) enrolled children with confirmed ASD as the index group and typically developing children as controls, a design that may inflate both sensitivity and specificity by excluding the diagnostically uncertain cases encountered in real-world screening. Second, review bias: in two studies (35, 39), the reference standard may have been influenced by prior knowledge of RITA-T results. In Choueiri et al. (35), the RITA-T scoring sheet accompanied the referral materials to the diagnostic clinic; in Kong et al. (39), the same examiners administered the screening and diagnostic measures and were not blinded to the findings, as acknowledged by the authors. By contrast, in Choueiri & Wagner (32), the reference-standard clinicians were blinded to the RITA-T results, and in Yassin et al. (36), the reference-standard diagnosis was based on the M-CHAT-R and ADOS-2 results without reference to the RITA-T. Third, applicability limitations: Richter et al. (44) was a training/implementation study that was excluded from the QUADAS-2 assessment (see Note above) because the tool was designed for diagnostic accuracy studies and does not fit training/implementation study designs. This distinction should be considered when interpreting the overall evidence base.

### Cross-cultural validation

RITA-T has been validated in five countries across three continents, with consistent psychometric performance across diverse populations.

In Canada, Lemay et al. (43) evaluated 239 children referred to an ASD diagnostic clinic after the RITA-T was integrated into the clinic’s screening and diagnostic assessment pathway. The study identified an optimal cutoff of 14, yielding sensitivity of 97%, specificity of 71%, PPV of 95%, and NPV of 79%. Notably, no child scoring below 12 received an ASD diagnosis. The authors proposed a risk stratification approach: scores below 12 indicate low risk (0% ASD diagnosis rate), scores of 12–16 indicate moderate risk (68.3%), and scores of 17 or above indicate high risk (97.7%). This stratified approach carries clinical utility for guiding referral decisions and parental counseling.

In the United States, Choueiri et al. (33) evaluated RITA-T in a culturally diverse community sample of 81 toddlers, confirming its strong psychometric properties in an underserved population. RITA-T scores were strongly associated with ADOS-G diagnostic indicators, and RITA-T screening was associated with shorter wait times for diagnostic evaluation.

In Lebanon, Yassin et al. (36) conducted a cross-cultural pilot study validating RITA-T in Arabic-speaking toddlers at the American University of Beirut Medical Center. The study included 48 toddlers aged 18–36 months and identified an optimal cutoff of 15, with sensitivity of 96%, specificity of 100%, PPV of 100%, and NPV of 96%. This was the first validation of RITA-T in an Arabic-speaking population and demonstrated that the tool maintained excellent psychometric properties following Arabic translation and cultural adaptation.

Kadak et al. (45) conducted the Turkish validation in a sample of 81 children aged 18–36 months. The Turkish version showed good sensitivity and specificity, confirming RITA-T’s cross-cultural applicability. The study highlighted that RITA-T could effectively discriminate ASD from non-ASD comparison groups, including language disorder and developmental delay, in a Turkish clinical population, supporting its use across diverse linguistic and cultural contexts.

The most recent cross-cultural adaptation was performed by Liu et al. (38), who translated RITA-T into Mandarin Chinese and validated it in a sample of 114 children. The Chinese version yielded an optimal cutoff of 16, with sensitivity of 0.918, specificity of 0.981, and AUC of 0.961 (95% CI: 0.924–0.998, p < 0.001). Internal consistency (Cronbach’s α = 0.828), test-retest reliability (r = 0.962), and inter-rater reliability (ICC = 0.975) were all satisfactory. The Chinese validation also demonstrated RITA-T’s capacity to differentiate among ASD, global developmental delay, and developmental language disorder, supporting its clinical utility in complex differential diagnosis.

The cross-cultural validation data are summarized in **Table 2**.

**Table 2:** Cross-cultural validation of RITA-T: diagnostic accuracy across populations.

Study	Country	Sample size	Optimal cutoff	Sensitivity	Specificity	AUC
Choueiri & Wagner (2015) (32)	USA	61	15	1.00	0.84	—†
Yassin et al. (2020) (36)	Lebanon	48	15	0.96	1.00	—†
Lemay et al. (2020) (43)	Canada	239	14	0.97	0.71	0.953(0.919–0.987)
Choueiri et al. (2021) (33)	USA	81	—	—	—	—†
Kong et al. (2021) (39)	USA	35	14	0.81	0.89	0.84 (0.691–0.993)
Kadak et al. (2024) (45)	Turkey	81	17	0.90	0.927	0.977 (0.919–0.987)
Liu et al. (2026) (38)	China	114	16	0.918	0.981	0.961(0.924–0.998)

†95% CI not reported in the original study. AUC = area under the receiver operating characteristic curve; CI, confidence interval.

### Clinical applications and community models

Choueiri et al. (33) expanded their work to a culturally diverse community sample of 81 toddlers in the United States. That study confirmed RITA-T’s strong psychometric properties and its utility in improving diagnostic access for underserved communities.

RITA-T has been implemented across several clinical settings. Lemay et al. (43) demonstrated its feasibility as a triage tool in a tertiary ASD diagnostic clinic, where the stratified scoring approach helped prioritize diagnostic resources for children at highest risk. Choueiri et al. (34) developed and evaluated a community-based RITA-T model in which community healthcare providers were trained to administer the instrument in primary care settings, with diagnostic confirmation provided by affiliated academic centers. Over the study period, this model significantly reduced the age at which children received ASD diagnoses and increased identification rates among minority and low-income families.

Choueiri et al. (35) further examined demographic and socioeconomic characteristics of children diagnosed through RITA-T screening and found that the tool identified ASD across diverse socioeconomic strata, suggesting that interactive, performance-based instruments may mitigate some disparities inherent in parent-report screening. These findings are consistent with broader research documenting persistent disparities in ASD detection among Black and Hispanic children (25, 26) and the role of systemic barriers in delaying diagnosis (27).

Training and scalability have also been investigated. Richter et al. (44) provided preliminary evidence that pediatric residents could learn to administer and interpret RITA-T after a structured training session, supporting the potential feasibility of RITA-T implementation within existing healthcare training frameworks. However, as this was a pilot implementation study, formal reliability statistics and long-term competency assessment were not reported.

Regarding global applicability, the World Health Organization has identified the development of culturally appropriate, low-cost screening tools as a priority for global autism detection (46). Systematic reviews of ASD screening in low- and middle-income countries (LMICs) have found that most screening tools require significant cultural adaptation and that workforce shortages pose major implementation barriers (46, 47). Task-sharing approaches, in which non-specialist healthcare workers are trained to administer screening tools, have been identified as a key strategy for LMIC settings (49). RITA-T’s brief administration time and minimal equipment requirements make it a promising candidate for such task-sharing models. The Chinese (38), Turkish (45), and Lebanese (36) validations provide initial evidence that RITA-T can be successfully adapted across diverse cultural and linguistic contexts, including health systems with varying resource levels. Implementation of universal screening in federally qualified health center sites has shown that structured protocols can significantly increase screening rates and referral to early intervention (50), suggesting models that could be adapted for RITA-T dissemination.

### Comparison with other screening tools

Compared with the M-CHAT-R/F, the most widely used level 1 ASD screener, RITA-T offers several potential advantages. M-CHAT-R/F is a parent questionnaire requiring 5–10 minutes with moderate pooled PPV of 57.7% (95% CI 48.6–66.8) in a recent meta-analysis (15). By contrast, RITA-T is clinician-administered and interactive, taking approximately 10 minutes with a reported PPV of 0.88 (derived from a single study in a referred sample). PPV estimates for the original M-CHAT in community-based settings ranged from 0.06 to 0.54 (13), reflecting the influence of population prevalence on predictive values; the M-CHAT-R/F demonstrates substantially higher pooled PPV of 57.7% (95% CI 48.6–66.8) in meta-analytic data (15). However, because RITA-T requires trained personnel, it is best positioned as a level 2 screener rather than a universal first-line tool. (**Table 3**).

**Table 3:** Comparison of M-CHAT-R/F and RITA-T screening instruments.

Feature	M-CHAT-R/F	RITA-T
Format	Parent questionnaire	Clinician-administered, interactive
Administration time	5–10 minutes	~10 minutes
Scoring	Parent-report questionnaire with a structured follow-up interview	Clinician observation
Age range	16–30 months	18–36 months
Level	1 (universal screening)	2 (targeted screening)

Among other level 2 screeners, the Screening Tool for Autism in Toddlers and Young Children (STAT) is a play-based, interactive instrument originally developed for 2-year-olds (51). It has demonstrated strong psychometric properties, including excellent inter-rater reliability, sensitivity of 0.92–0.95, and specificity of 0.73–0.85 in children aged 24–36 months (52). Subsequent research has extended its use to children under 24 months (53) and validated it for primary care settings (54). Separate scoring algorithms have been developed to optimize STAT for different screening priorities (55), and its classification shows strong concordance with ADOS-2 diagnosis (56). Implementation research has demonstrated sustained fidelity in early intervention programs (57). The STAT, however, requires approximately 20 minutes to administer, making it less feasible for routine well-child visits compared with the 10-minute RITA-T.

The Autism Detection in Early Childhood (ADEC) is another clinician-administered level 2 screening tool for children under 3 years. The original validation demonstrated high internal consistency (α = 0.91) and concurrent validity with ADOS and ADI-R, with sensitivity of 1.0 and specificity of 0.74–0.90 at optimal cutoff (58). US validation confirmed good sensitivity (0.93–0.94) and acceptable specificity (0.62–0.64) in a clinically referred sample (59), and cross-cultural adaptation in Mexico demonstrated sensitivity of 0.79–0.94 and specificity of 0.88–1.00 (60). The ADEC has also shown promise in predicting long-term outcomes (61), and a telehealth adaptation (ADEC-V) has been validated (62). However, the ADEC requires specialized materials and a longer administration protocol than RITA-T.

Compared with these instruments, RITA-T offers the shortest administration time (approximately 10 minutes versus 15–20 minutes for ADEC and approximately 20 minutes for STAT), which is a practical advantage in busy clinical settings. In terms of diagnostic accuracy, RITA-T sensitivity (0.74–1.00) is comparable to ADEC (0.79–1.00) and slightly lower than STAT (0.92–0.95), though direct comparisons are limited by differences in reference standards and population characteristics across studies. Specificity ranges overlap considerably: RITA-T (0.50–1.00), STAT (0.73–0.85), and ADEC (0.62–1.00), reflecting the influence of cutoff selection and sample composition rather than fundamental differences in discriminative ability. RITA-T requires only basic toys and a picture book, whereas STAT and ADEC both require standardized testing kits that add to material cost and logistics. Training requirements differ: RITA-T is administered after structured training, STAT requires play-based observation training, and ADEC requires standardized administration protocols. A notable limitation of RITA-T compared with STAT and ADEC is the absence of a validated telehealth version; both STAT and ADEC-V have demonstrated feasibility for remote administration, which may be important for expanding access in underserved areas. PPV was reported only in the original RITA-T study (0.88), and was not consistently reported across STAT or ADEC validation studies, limiting cross-tool PPV comparison. While RITA-T requires minimal specialized materials compared with other instruments, **Table 4** provides a qualitative comparison of material requirements, which should be interpreted with caution as no formal cost-effectiveness analysis was conducted. (**Table 4**).

**Table 4:** Comparison of level 2 screening tools for autism spectrum disorder in toddlers.

Feature	RITA-T	STAT	ADEC
Administration time	~10 min	~20 min	~15–20 min
Age range	18–36 months	12–36 months	18–36 months
Number of items	9	12	16
Cross-cultural validations	US, Canada, Lebanon, Turkey, China	US	US, Mexico
Telehealth version	No validated telehealth version of RITA-T was identified in the studies included in this review.	Yes	Yes (ADEC-V)
Sensitivity range	0.74-1.00	0.92-0.95	0.79-1.00
Specificity range	0.50–1.00	0.73-0.85	0.62-1.00
Training requirements	Clinician-administered after structured training	Clinician-administered with play-based observation training	Clinician-administered with standardized materials
Materials needed	Toys and picture book	Standardized toys and testing kit	Standardized picture cards and testing kit

Sensitivity and specificity ranges are descriptive cross-study ranges and do not represent results of head-to-head comparisons. Differences in study populations, reference standards, and cutoff thresholds limit direct comparability across instruments.

### Telehealth and digital adaptations

The COVID-19 pandemic accelerated the development of telehealth-based autism screening and assessment. The teleNIDA, a videoconference-based screening instrument, has demonstrated good concurrent validity with in-person M-CHAT screening (63). Telehealth administration of the STAT has shown comparable accuracy to in-person assessment (64), and the TELE-ASD-PEDS has demonstrated strong validity and high caregiver satisfaction in remote assessment (65). The ADEC-V has been validated for telehealth evaluations (62), and telemedicine-based preliminary screening using multiple tools has effectively identified ASD risk in Chinese toddlers (66). To date, no telehealth adaptation of RITA-T has been formally validated. Given the successful adaptation of other interactive screening tools for remote administration, developing a telehealth version of RITA-T represents a high-priority direction that could substantially expand access for rural, underserved, and remote populations.

## Discussion

### Summary of evidence

This scoping review synthesized 10 original studies on the RITA-T, including 7 diagnostic validation studies encompassing 659 toddlers across five countries. The available evidence indicates that RITA-T is a promising level 2 screening instrument for ASD in toddlers aged 18–36 months, with consistent psychometric performance across English, Arabic, Turkish, and Chinese adaptations. Reported cutoff scores ranged from 14 to 17, with sensitivity of 0.74–1.00 and specificity of 0.50–1.00 across populations and reference standards. RITA-T possesses several practical advantages, including a brief 10-minute administration time, minimal equipment requirements, and the ability to discriminate ASD from other developmental conditions, though the underlying studies varied in population characteristics and reference standards.

### Quality of evidence and sources of heterogeneity

The observed heterogeneity in diagnostic accuracy warrants careful interpretation. Sensitivity and specificity varied considerably across studies, likely reflecting differences in sample composition, reference standard selection, and cutoff thresholds. Two early studies used case-control designs with known ASD cases and typically developing controls (32, 36), which tend to overestimate diagnostic accuracy relative to consecutive clinical cohorts (spectrum bias). Consistent with this pattern, these studies reported the highest sensitivity and specificity values. By contrast, studies using clinically referred or consecutive samples (38, 43, 45) yielded more conservative but likely more generalizable estimates. The four most recent validation studies (33, 34, 38, 45), which demonstrated no high risk of bias on QUADAS-2, provide the most reliable evidence on RITA-T’s performance in real-world settings.

A notable methodological consideration is that 5 of the 10 included studies were authored or co-authored by the tool’s developer (Choueiri and colleagues) (32–36). Although these studies generally adhered to rigorous methodology and several employed independent reference standard assessors, the concentration of evidence from a single research group underscores the need for independent replication by researchers without involvement in RITA-T’s development. Such replication is particularly important given that developer-led validation studies in diagnostic research have been associated with larger effect estimates than independent studies.

### Comparison with existing literature

The psychometric performance of RITA-T is comparable with other validated level 2 screening instruments. STAT has demonstrated sensitivity of 0.92–0.95 and specificity of 0.73–0.85 in children aged 24–36 months (52), while ADEC has shown sensitivity of 0.79–1.00 and specificity of 0.62–1.00 depending on the population (58–60). RITA-T’s reported accuracy ranges are broadly comparable, with considerable overlap, and RITA-T offers the additional advantage of a shorter administration time (approximately 10 minutes versus 15–20 minutes for STAT and ADEC). However, both comparator tools have been validated across broader age ranges (STAT: 12–36 months) and have established telehealth adaptations, representing gaps in the current RITA-T evidence base. The cross-cultural breadth of RITA-T validation (five countries across three continents) compares favorably with STAT and ADEC, suggesting that RITA-T may be particularly suitable for international dissemination efforts.

The available evidence also suggests that RITA-T addresses some limitations of parent-report screening instruments. Preliminary evidence from community implementation studies suggests that RITA-T-based screening may reduce disparities in ASD detection across socioeconomic strata (35), although these findings require replication in larger, more diverse populations.

### Limitations of the evidence base and this review

Several limitations constrain the conclusions of this review. First, the RITA-T literature remains limited in volume, with only 10 original studies and 659 toddlers in diagnostic validation samples. No studies have examined predictive validity against long-term developmental outcomes, which is essential for establishing the clinical utility of any screening instrument. Second, RITA-T has been validated only for children aged 18–36 months, although preliminary data suggest possible extension to older children (39). Third, the absence of a universally standardized certification program raises questions about cross-site fidelity as the tool is adopted more broadly. Fourth, the lack of a validated telehealth version limits applicability in remote and underserved settings. Fifth, no independent cost-effectiveness analysis of RITA-T-based screening has been conducted, although universal ASD screening has been shown to be cost-effective (29). Sixth, none of the included studies reported structural validity indicators (EFA, CFA, or model fit indices such as RMSEA, SRMR, CFI, TLI), precluding evaluation of whether RITA-T’s nine items load onto theoretically meaningful dimensions. This gap originates from the original development study (32), which did not derive explicit factor structures, and should be addressed in future validation work through hypothesis-driven CFA with COSMIN-guided fit criteria and cross-cultural measurement invariance testing.

At the review level, several limitations should be acknowledged. Despite a systematic search across five databases, unpublished studies and gray literature were not systematically sought, and language restrictions (English and Chinese only) may have excluded relevant studies in other languages. The review was not prospectively registered, which may introduce reporting bias. Moreover, half of the included studies were authored or co-authored by the tool’s developer (Choueiri and colleagues), which may introduce developer bias in the interpretation of psychometric data. The quality assessment relied on QUADAS-2, which was designed for diagnostic test accuracy studies and may not fully capture the methodological features of implementation and training studies included in this review. Finally, the relatively small number of studies precluded quantitative synthesis and formal assessment of publication bias.

### Implications for clinical practice and future research

The available evidence supports the use of RITA-T as a level 2 screening tool in settings where trained clinicians are available. Its brief administration time and minimal equipment requirements make it suitable for integration into community-based screening models. For clinical practice, the risk stratification approach proposed by Lemay et al. (43) provides a practical framework for triaging referral decisions: children scoring below 12 can be considered at low risk, those scoring 12–16 at moderate risk, and those scoring 17 or above at high risk for ASD. This stratified approach may help prioritize diagnostic resources for children most likely to receive an ASD diagnosis.

Several research priorities emerge from this synthesis. Large-scale, multi-center normative studies are needed to establish RITA-T norms across diverse populations. Independent replication studies conducted by researchers not involved in the tool’s development would strengthen the evidence base. Formal validation for children outside the current 18–36 month age range would extend the tool’s clinical utility. Longitudinal follow-up studies are necessary to examine RITA-T’s ability to predict later diagnostic and developmental outcomes. The development of tablet-based or digital versions could streamline administration, scoring, and data management, while direct head-to-head comparisons with other level 2 screeners would provide valuable comparative effectiveness data. A telehealth adaptation is particularly warranted, building on successful remote versions of STAT (64), ADEC (62), and TELE-ASD-PEDS (65). Formal economic evaluation of RITA-T-based screening pathways, compared with standard care, would inform implementation decisions. From an implementation perspective, standardized online training modules and certification programs would help ensure fidelity across sites, and cross-cultural validation should be expanded to low- and middle-income countries through task-sharing models (46–48) and to additional linguistic and cultural contexts beyond the current five countries.

## Conclusion

This scoping review indicates that RITA-T is an effective level 2 screening tool for ASD in toddlers aged 18–36 months. As a clinician-administered, interactive instrument, it addresses key limitations of parent-report methods by providing an objective, performance-based assessment of early social-communicative markers. Across five countries and 659 toddlers in diagnostic validation studies, RITA-T has consistently yielded strong psychometric properties, with reported cutoff scores of 14–17, sensitivity of 0.74–1.00, and specificity of 0.50–1.00. The evidence supports RITA-T as a tool that can improve the accuracy and timeliness of ASD detection, particularly when integrated into community-based screening models. Its 10-minute administration time and minimal material requirements offer practical advantages over STAT and ADEC. Future work should focus on independent replication, expanding normative data, validating extended age ranges, developing telehealth and digital platforms, and conducting comparative effectiveness and cost-effectiveness analyses. The growing body of international validation studies underscores RITA-T’s potential for broader global application in early ASD screening.

## Data Availability

The original contributions presented in the study are included in the article/**Supplementary Material**. Further inquiries can be directed to the corresponding author.

## Appendix A: PubMed Search Strategy

A systematic search of PubMed was conducted on June 15, 2026, using the following query:

((“Rapid Interactive Screening Test for Autism in Toddlers”[Title/Abstract]) OR (“RITA-T”[Title/Abstract])) AND ((“autism spectrum disorder”[MeSH Terms]) OR (“autism”[Title/Abstract]) OR (“ASD”[Title/Abstract]) OR (“screening”[Title/Abstract]) OR (“early diagnosis”[Title/Abstract]) OR (“early detection”[Title/Abstract])) AND ((“2015/01/01”[Date - Publication]: “2026/06/15”[Date - Publication])) AND (english[Language] OR chinese[Language])

The search yielded 31 records. This strategy was adapted for Web of Science, Scopus, PsycINFO (via Ovid), and CNKI (China National Knowledge Infrastructure) using equivalent controlled vocabulary and syntax. No additional limits were applied beyond language (English or Chinese) and publication date (January 2015 through June 2026). Reference lists of included studies and related reviews were hand-searched for additional records, yielding 17 additional records (total 48 before deduplication).
