# Supplementary material for: RITA-T (rapid interactive screening test for autism in toddlers): a scoping review of psychometric properties, cross-cultural validation, and clinical applications
Source: Front Psychiatry. 2026 Aug 27;17:1922791. doi: 10.3389/fpsyt.2026.1922791 (PMC13559430; doi:10.3389/fpsyt.2026.1922791)
Supplement: Supplementary file 1 [file Table1.docx]

**PRISMA-ScR Checklist**

*Preferred Reporting Items for Systematic reviews and Meta-Analyses extension for Scoping Reviews*

| **Section** | **Item** | **PRISMA-ScR Checklist Item** | **Location** |
| --- | --- | --- | --- |
| Title | 1 | Identify the report as a scoping review. | Title page |
| Abstract | 2 | Provide a structured summary including background, objective, methods, results, and conclusions. | Abstract |
| Introduction | 3 | Describe the rationale for the review in the context of existing knowledge. | Introduction |
|  | 4 | Provide an explicit statement of the questions and objectives. | Abstract (Objective); Introduction (final paragraph) |
| Methods | 5 | Indicate whether a review protocol exists. | Methods, para 1 |
|  | 6 | Specify characteristics of sources used as eligibility criteria. | Methods: Inclusion and Exclusion Criteria |
|  | 7 | Describe all information sources and date last searched. | Methods: Search Strategy; Appendix A |
|  | 8 | Present full electronic search strategy for at least one database. | Appendix A |
|  | 9 | State the process for selecting sources of evidence. | Methods: Study Selection and Data Extraction |
|  | 10 | Describe data charting methods (piloted forms, independent reviewers). | Methods: Study Selection and Data Extraction |
|  | 11 | List and define all variables for which data were sought. | Methods: Study Selection and Data Extraction |
|  | 12 | Describe methods for assessing risk of bias of individual sources. | Methods: Quality Assessment (QUADAS-2) |
|  | 13 | Describe methods of handling and summarizing the data. | Methods: Study Selection and Data Extraction |
| Results | 14 | Numbers of studies screened, assessed for eligibility, and included with reasons. | Results: Study Selection; Figure 1 |
|  | 15 | For each source, present characteristics and provide citations. | Results: Study Characteristics; Cross-Cultural Validation table |
|  | 16 | Present data on risk of bias of each source. | Results: Quality Assessment of Included Studies |
|  | 17 | For each included source, present relevant data on each key variable. | Results: all thematic subsections |
|  | 18 | Summarize and present the results of the synthesis. | Results; Discussion: Summary of Evidence |
| Discussion | 19 | Summarize the main results and relevance to objectives. | Discussion: Summary of Evidence |
|  | 20 | Discuss limitations of the review process and evidence. | Discussion: Limitations of the Evidence Base |
|  | 21 | Provide general interpretation with implications. | Discussion: Implications; Conclusion |
| Funding | 22 | Describe sources of funding and role of funders. | Funding |

*Reference: Tricco AC, Lillie E, Zarin W, et al. PRISMA extension for scoping reviews (PRISMA-ScR): checklist and explanation. Ann Intern Med. 2018;169(7):467-473. doi:10.7326/M18-0850*
