# Supplementary material for: RITA-T (rapid interactive screening test for autism in toddlers): a scoping review of psychometric properties, cross-cultural validation, and clinical applications
Source: Front Psychiatry. 2026 Aug 27;17:1922791. doi: 10.3389/fpsyt.2026.1922791 (PMC13559430; doi:10.3389/fpsyt.2026.1922791)
Supplement: Supplementary file 2 [file Table2.docx]

**Supplementary Materials**

*RITA-T (Rapid Interactive Screening Test for Autism in Toddlers):A Scoping Review of Psychometric Properties, Cross-Cultural Validation, and Clinical Applications*

# Supplementary File 1: Complete Search Strategy

The following search strategies were developed and documented prior to commencing the literature search. Searches were conducted across five databases from database inception through June 15, 2026.

## 1.1 PubMed

(("Rapid Interactive Screening Test for Autism in Toddlers"[Title/Abstract]) OR ("RITA-T"[Title/Abstract])) AND (("autism spectrum disorder"[MeSH Terms]) OR ("autism"[Title/Abstract]) OR ("ASD"[Title/Abstract]) OR ("screening"[Title/Abstract]) OR ("early diagnosis"[Title/Abstract]) OR ("early detection"[Title/Abstract])) AND (("2015/01/01"[Date - Publication] : "2026/06/15"[Date - Publication])) AND (english[Language] OR chinese[Language])

This search yielded 31 records. The strategy was adapted for Web of Science, Scopus, PsycINFO (via Ovid), and CNKI (China National Knowledge Infrastructure) using equivalent controlled vocabulary and syntax. No additional limits were applied beyond language (English or Chinese) and publication date (January 2015 through June 2026). Reference lists of included studies and related reviews were hand-searched for additional records, yielding 17 additional records (total 48 before deduplication).

## 1.2 Web of Science

TS=("RITA-T" OR "Rapid Interactive Screening Test for Autism in Toddlers") AND TS=("autism" OR "autism spectrum disorder" OR "ASD" OR "screening" OR "early diagnosis" OR "early detection") AND PY=(2015-2026)

## 1.3 Scopus

TITLE-ABS-KEY("RITA-T" OR "Rapid Interactive Screening Test for Autism in Toddlers") AND TITLE-ABS-KEY("autism" OR "autism spectrum disorder" OR "ASD" OR "screening" OR "early diagnosis" OR "early detection") AND PUBYEAR > 2014 AND PUBYEAR < 2027 AND LANGUAGE(english OR chinese)

## 1.4 PsycINFO (via Ovid)

(DE "Autism Spectrum Disorders" OR DE "Pervasive Developmental Disorders" OR DE "Screening") AND (TI "RITA-T" OR AB "RITA-T" OR TI "Rapid Interactive Screening Test" OR AB "Rapid Interactive Screening Test")

## 1.5 China National Knowledge Infrastructure (CNKI)

SU=("RITA-T" OR "快速互动式幼儿自闭症筛查测试") AND SU=("自闭症" OR "孤独症" OR "筛查" OR "早期识别")

## 1.6 Hand-Searching

Reference lists of all included studies and related review articles were manually searched for additional eligible records not captured by the database searches, yielding 17 additional records (total 48 before deduplication).

## Supplementary File 2: Eligibility Criteria (PICOS Framework)

The following eligibility criteria were defined a priori and documented prior to commencing the search.

| Criterion | Inclusion | Exclusion |
| --- | --- | --- |
| Population | Toddlers aged 18-36 months | Children outside 18-36 month age range without RITA-T data |
| Index Test | RITA-T (Rapid Interactive Screening Test for Autism in Toddlers) | Other screening instruments without RITA-T administration |
| Reference Standard | ADOS-2, ADOS-G, DSM-5 clinical diagnosis, or multidisciplinary team evaluation | No reference standard or non-standard diagnostic procedures |
| Study Design | Original empirical studies reporting psychometric properties (sensitivity, specificity, PPV, NPV, AUC, reliability) | Reviews, editorials, conference abstracts, case reports, commentaries |

Studies were included if they (a) reported original empirical data on RITA-T administration in toddlers aged 18-36 months, (b) included a comparison with a reference standard, (c) reported at least one psychometric property, and (d) were published in English or Chinese.

## Supplementary File 3: Data Extraction Framework

A standardized data extraction form was developed and piloted on three randomly selected studies before full extraction. The following data items were extracted from each included study:

### 3.1 Study Characteristics

- Author(s) and year of publication
- Country/region of study conduct
- Study design (prospective, retrospective, cross-sectional)
- Sample size (total, ASD group, comparison groups)
- Age range of participants
- Setting (clinic, community, hospital)

### 3.2 RITA-T Administration Details

- Cutoff score used
- Administrator training requirements
- Language/version of RITA-T administered
- Administration time reported

### 3.3 Psychometric Properties

- Sensitivity (95% CI if reported)
- Specificity (95% CI if reported)
- Positive Predictive Value (PPV)
- Negative Predictive Value (NPV)
- Area Under the Curve (AUC) with 95% CI
- Internal consistency (Cronbach's alpha)
- Test-retest reliability (correlation coefficient)
- Inter-rater reliability (ICC)

### 3.4 Reference Standard

- Reference standard used (ADOS-2, ADOS-G, DSM-5, MDT)
- Reference standard assessor blinding to RITA-T results
- Time interval between RITA-T and reference standard

### 3.5 Quality Assessment

- QUADAS-2 risk of bias ratings (4 domains)
- QUADAS-2 applicability concerns (3 domains)
- Methodological notes and specific concerns

## Supplementary File 4: Data Synthesis Plan

Given the anticipated heterogeneity in study designs, populations, reference standards, and cutoff thresholds, a narrative synthesis approach was adopted rather than quantitative meta-analysis. The synthesis was structured around the following themes:

- Psychometric properties across studies (sensitivity, specificity, PPV, NPV, AUC)
- Cross-cultural validation outcomes (comparison of performance across language versions)
- Comparison with other level 2 screening instruments (STAT, ADEC)
- Clinical application models and implementation feasibility
- Methodological quality and risk of bias patterns

Where studies reported sufficient statistical detail, 95% confidence intervals were extracted and presented in summary tables. Sources of heterogeneity were explored narratively, including differences in sample composition, reference standard selection, cutoff thresholds, and study design.

## Supplementary File 5: PRISMA-ScR Checklist

The completed PRISMA-ScR (Preferred Reporting Items for Systematic Reviews and Meta-Analyses extension for Scoping Reviews) checklist is provided as a separate supplementary file.

# Supplementary File 6: Full QUADAS-2 Assessment of Included Studies

The complete QUADAS-2 assessment is reported below in accordance with the tool's domain structure. Signaling questions are answered as Yes, No, or Unclear; domain-level risk-of-bias and applicability judgments are reported as Low, High, or Unclear. The flow-and-timing domain does not include an applicability judgment in QUADAS-2. The overall applicability summary presented in Table 1 of the main text is an author-derived synthesis, defined as the most conservative rating across the three domains that include an applicability assessment, and it supplements rather than replaces the domain-specific judgments reported below.

Supplementary Table 1. Full QUADAS-2 assessment of included studies.

| **QUADAS-2 domains and signaling questions** | **Choueiri & Wagner (2015) (32)** | **Yassin et al. (2020) (36)** | **Lemay et al. (2020) (43)** | **Choueiri et al. (2021) (33)** | **Kong et al. (2021) (39)** | **Choueiri et al. (2023) (34)** | **Choueiri et al. (2024) (35)** | **Kadak et al. (2024) (45)** | **Liu et al. (2026) (38)** | **Richter et al. (2026) (44)** |
| --- | --- | --- | --- | --- | --- | --- | --- | --- | --- | --- |
| **Domain 1: Patient selection** |  | | | | | | | | | |
| Was a consecutive or random sample of patients enrolled? | Unclear | Unclear | Yes | Unclear | Unclear | Yes | Yes | Unclear | Unclear | N/A |
| Was a case-control design avoided? | No | No | Yes | Yes | Unclear | Yes | Yes | Yes | Unclear | N/A |
| Did the study avoid inappropriate exclusions? | Yes | Yes | Yes | Yes | Unclear | Yes | Yes | Yes | Yes | N/A |
| Risk-of-bias judgment (Domain 1) | High | High | Low | Low | Unclear | Unclear | High | Unclear | Unclear | N/A |
| Applicability concern: Is there concern that the included patients do not match the review question? | High | High | Low | Low | High | Unclear | Unclear | High | Unclear | N/A |
| **Domain 2: Index test** |  | | | | | | | | | |
| Were the index test results interpreted without knowledge of the results of the reference standard? | Yes | Yes | Yes | Yes | Yes | Yes | Yes | Yes | Yes | N/A |
| If a threshold was used, was it pre-specified? | Unclear | Unclear | Unclear | Unclear | Unclear | Unclear | Unclear | Unclear | Unclear | N/A |
| Risk-of-bias judgment | Low | Low | Low | Low | Low | Low | Low | Low | Low | N/A |
| Applicability concern: Is there concern that the index test, its conduct, or interpretation differs from the review question? | Low | Low | Low | Low | Low | Low | Low | Low | Low | N/A |
| **Domain 3: Reference standard** |  | | | | | | | | | |
| Is the reference standard likely to correctly classify the target condition? | Yes | Yes | Yes | Yes | Yes | Yes | Yes | Yes | Yes | N/A |
| Were the reference standard results interpreted without knowledge of the results of the index test? | Yes | Yes | Unclear | Yes | No | Unclear | No | Yes | Unclear | N/A |
| Risk-of-bias judgment (Domain 3) | Low | Low | Unclear | Low | High | Unclear | High | Low | Unclear | N/A |
| Applicability concern: Is there concern that the target condition as defined by the reference standard does not match the review question? | Low | Low | Low | Low | Low | Low | Low | Low | Low | N/A |
| **Domain 4: Flow and timing** |  | | | | | | | | | |
| Was there an appropriate interval between index test(s) and reference standard? | Unclear | Yes | Unclear | Unclear | Unclear | Unclear | Unclear | Yes | Unclear | N/A |
| Did all patients receive a reference standard? | Yes | Yes | Yes | Yes | Yes | Yes | Yes | Yes | Yes | N/A |
| Did patients receive the same reference standard? | Yes | Yes | Yes | Yes | Yes | Yes | Yes | Yes | Yes | N/A |
| Were all patients included in the analysis? | Yes | Yes | Yes | Yes | Yes | Yes | Yes | Yes | Yes | N/A |
| Risk-of-bias judgment | Low | Low | Low | Low | Low | Unclear | Unclear | Low | Low | N/A |
| Applicability concern | Not applicable in QUADAS-2 | Not applicable in QUADAS-2 | Not applicable in QUADAS-2 | Not applicable in QUADAS-2 | Not applicable in QUADAS-2 | Not applicable in QUADAS-2 | Not applicable in QUADAS-2 | Not applicable in QUADAS-2 | Not applicable in QUADAS-2 | Not applicable in QUADAS-2 |
